# Observation and quantification of the quantum dynamics of a strong-field excited multi-level system

**DOI:** 10.1038/srep39993

**Published:** 2017-01-04

**Authors:** Zuoye Liu, Quanjun Wang, Jingjie Ding, Stefano M. Cavaletto, Thomas Pfeifer, Bitao Hu

**Affiliations:** 1 School of Nuclear Science and Technology, Lanzhou University, 730000, China; 2 Max-Planck-Institut für Kernphysik, Heidelberg, 69117, Germany

## Abstract

The quantum dynamics of a V-type three-level system, whose two resonances are first excited by a weak probe pulse and subsequently modified by another strong one, is studied. The quantum dynamics of the multi-level system is closely related to the absorption spectrum of the transmitted probe pulse and its modification manifests itself as a modulation of the absorption line shape. Applying the dipole-control model, the modulation induced by the second strong pulse to the system’s dynamics is quantified by eight intensity-dependent parameters, describing the self and inter-state contributions. The present study opens the route to control the quantum dynamics of multi-level systems and to quantify the quantum-control process.

Absorption of light is one of the most fundamental optical properties of a material. Based on the pump-probe technique, transient-absorption spectroscopy (TAS) becomes one of the most powerful experimental tools for a “fingerprint” to identify atomic and molecular species and understand the related dynamics^1^,^2^,^3^. In contrast to schemes based on electron spectra, bound-state transitions can be accessed by TAS even in the absence of ionization. In recent transient-absorption measurements, the quantum dynamics of different molecules were studied on the femtosecond time scale^4^,^5^,^6^,^7^, and the observation of electron dynamics on their natural time scale was achieved by employing extreme ultraviolet (XUV) attosecond pulses^8^.

Thus far, however, researchers’ attentions have mainly been focused on the pump-probe side, i.e., a quantum system is initially excited by a short pump pulse and its time-dependent evolution is probed by a subsequently arriving probe pulse via measurement of its absorption spectrum. This enabled, e.g., quantum coherent control of photo-induced dynamics by manipulating interferences among different coherent excitation pathways with ultrashort laser pulses^9^,^10^,^11^,^12^,^13^, and the understanding of the dynamics of complex control processes^14^,^15^. Novel studies with inverted arrival order have recently attracted considerable attention^16^,^17^,^18^,^19^,^20^,^21^. There, a quantum system is coherently excited by the probe pulse arriving first, and the delayed pump pulse manipulates the system’s dynamics, giving rise to a modulation of the probe’s absorption spectrum known as perturbed polarization decay^19^,^20^, most recently also Fano phase control^22^. The possibility to control the absorption properties of an atomic system by an induced phase shift due to a laser pulse is a promising result. However, it is still an open question how one can transfer this control concept to fully understand and manipulate the spectral response of a multi-electron atom^23^,^24^ or molecule^25^,^26^,^27^,^28^ interacting with strong fields.

Here, connecting the absorption line shape to the quantum dynamics of a V-type three level system, the dipole-control model (DCM) is used to quantify absorption line-shape changes induced by the pump pulse, by taking into account the self and inter-state contributions as introduced for the case of doubly-excited Helium^18^. Taking the multi-level system (the ground state 5 s ^2^S_1/2_ and the fine-structure excited states 5p ^2^P_1/2,3/2_) of atomic rubidium as a sample, we performed transient-absorption measurements with femtosecond laser pulses. By applying the DCM, this pump-intensity-dependent modulation is quantified in terms of eight parameters, which are extracted from the experimental absorption spectrum.

## General idea

The evolution of the atomic system is here described via the density matrix 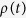. An off-diagonal element 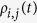 (*i* ≠ *j*) represents the coherence between states |*i*〉 and |*j*〉, while a diagonal element 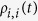 quantifies the population of level |*i*〉 at energy *ω*_*i*_. For the case of a single resonance |1〉 → |2〉 interacting only with a weak probe pulse, the absorption spectrum *S*_ab_(*ω*) is related to the time evolution of the off-diagonal matrix element *ρ*_12_(*t*) as 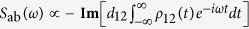
29,30, which gives rise to a Lorentzian absorption line centered on the transition energy. The off-diagonal matrix element is given by 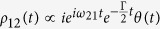, such that the generated time-dependent dipole response results from *d*_12_*ρ*_12_(*t*). Here, *ω*_21_, *d*_12_ and Γ denote the energy, the dipole-moment matrix element and the decay width of the resonance, respectively, and *θ* (*t*) is the Heaviside function. In the presence of a subsequent strong-field (pump) laser pulse, the evolution of the excited quantum system is modified, such that the modification in the system’s dipole response results in a change of the absorption line shape^22^. In the following, we call this pump pulse a control pulse, because its action is more general than just a single-photon excitation. The modification of the absorption line shape can be understood as the result of a complex time-delay-dependent amplitude and phase change *A*(*τ*) induced by the control pulse to the dipole response of the system generated by the probe pulse^31^. The spectral response as a function of the probe-control delay *τ* is given by 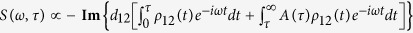. The form of *A*(*τ*) is determined by the interaction process. Self-processes, i.e., processes such as AC Stark shift, strong-field ionization, or Rabi oscillations which do not involve other nearby coherently excited states, can be modeled by 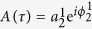, with 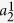 describing an amplitude change of the system’s dipole response and 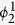 representing a phase change. Both parameters depend on the control-pulse intensity. On the other hand, a time-delay-dependent amplitude and phase modification 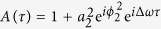, with respect to the probe-control delay *τ*, applies for inter-state processes associated with the coupling to another coherently excited state, where Δ*ω* is the energy difference between the two states. 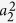 and 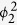 denote the amplitude and phase parameters, respectively, which also depend on the control-pulse intensity.

**Figure 1:**
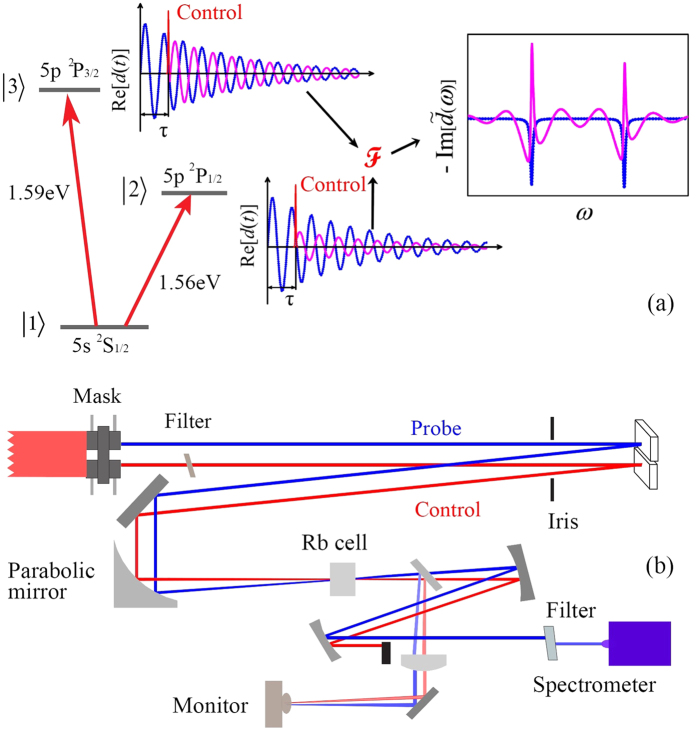
(**a**) The diagram of the V-type three-level system in Rb (*ω*_21_ = 1.56 eV, *ω*_31_ = 1.59 eV)^36^ excited and driven by two ultrashort broadband laser pulses, the amplitude and phase modification of the system’s dipoles at arbitrary time delay *τ* induced by the control pulse, and the spectral response of the system. (**b**) Illustrative sketch of the experimental setup.

For the V-type three-level system shown in Fig. 1(a), interacting with a weak probe pulse, the absorption spectrum exhibits two Lorentzian absorption lines centered on the transition energies. In the presence of a control pulse, the system’s dipole response will be affected by inserting a complex factor *A*_*k*_(*τ*), as illustrated in Fig. 1(a). The spectral response with respect to the probe-control delay *τ* is given by


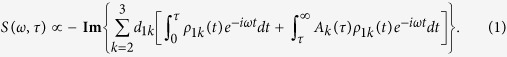


Here, *d*_12_ and *d*_13_ are the dipole moment matrix elements of both transitions, and the general spectral response is shown by the pink line of the inset in Fig. 1(a). Since both the self-process and inter-state process induced by the control pulse contribute to the manipulation of the absorption line shape^14^,^24^,^32^, the general complex amplitude and phase modification *A*_*k*_(*τ*) is given by


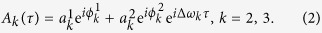


Here, Δ*ω*_2_ = *ω*_31_ − *ω*_21_ and Δ*ω*_3_ = −Δ*ω*_2_. The DCM parameters of the self-processes describe how the value of a coherence *ρ*_1*k*,fin_ after interaction with an intense pulse depends on the value of that same coherence *ρ*_1*k*,in_ before interaction with the pulse. Since both of them are equally affected by a change in the time delay *τ*, these parameters appear as a time-delay-independent contribution in the transformation functions *A*_*k*_(*τ*). In contrast, the DCM parameters of inter-state processes quantify how the final value of a coherence *ρ*_1*k*,fin_ (k = 2, 3) after interaction with an intense pulse depends on the value of the other coherence before interaction. By changing the time delay *τ*, the two coherences oscillate at different frequencies *ω*_*k*1_, giving rise to a slowly oscillating contribution in *A*_*k*_(*τ*) at the frequency difference Δ*ω*_*k*_.

The decay rates of both excited states Γ_2_ and Γ_3_ used to model the results with the DCM are much larger than the spontaneous decay rate, and effectively account for Doppler broadening and collision-induced broadening, as well as for the additional broadening in the nanosecond pedestal of the femtosecond pulses, which depends on the control pulse intensity. In the following, the decay rates are treated as free fitting parameters extracted from the experimental measurements. These decay times are much longer than the interaction time, such that the modulation induced by the femtosecond control pulse is not significantly sensitive to Γ_*k*_ and can be effectively modeled by the DCM. The intrinsic sensitivity of the transient-absorption measurement to the quantum dynamics of the system manifests itself via sharp spectroscopic signatures, apparent in the time-delay-dependent spectral line shapes. The quantum dynamical changes of this multi-level system are here included via eight intensity-dependent modulation parameters extracted from the absorption spectrum.

## Results and Discussions

**Figure 2:**
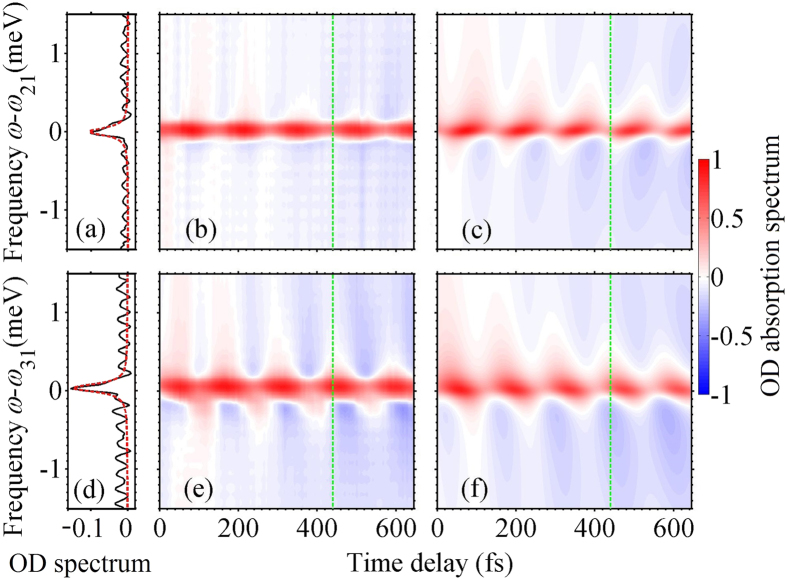
Experimental (black solid line) and simulated (red dashed line) absorption spectra (**a**) *S*_0_(*ω* − *ω*_21_) and (**d**) *S*_0_(*ω* − *ω*_31_) around the transition energies *ω*_21_ = 1.56 eV and *ω*_31_ = 1.59 eV, respectively, of a probe pulse of intensity *I*_pr_ = 3.0 × 10^8^ W/cm^2^, transmitted through an ensemble of Rb atoms. Panels (b) and (e) show *S*(*ω* − *ω*_21_, *τ*) and *S*(*ω* − *ω*_31_, *τ*) with a control pulse of intensity *I*_co_ = 2.13 × 10^10 ^W/cm^2^ with varying time delay, while panels (c) and (f) show the corresponding theoretical results by applying the DCM. Results indicated by the vertical dashed lines with a time delay of 440 fs in (**b**) and (**e**) correspond to the absorption spectrum lined out in Fig. 3(a), while these in (**c**) and (**f**) correspond to the absorption spectrum that lined out in Fig. 3(b).

In order to test the DCM and apply it to quantify the dynamical changes of a quantum system, we perform transient-absorption measurements with ultrashort laser pulses acting as control and probe pulses. The transient-absorption setup is illustrated in Fig. 1(b). The measured optical density (OD) absorption spectrum is given by 
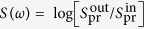
, where 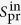 and 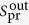 are the energy spectra of the probe pulse before and after interacting with the rubidium gas, respectively. The weak probe pulse, arriving first, excites the quantum system by driving population from the ground state to the upper two excited states through single-photon excitation. The quantum system is then affected by the subsequently arriving control pulse, and the related dynamical information is imprinted into the probe absorption line shape, which is experimentally measured. With a fixed probe-pulse intensity of *I*_pr_ = 3.0 × 10^8^ W/cm^2^, results in Fig. 2 show the absorption spectra of the probe pulse transmitted through the rubidium sample. The absorption spectra at the transition energies *ω*_21_ and *ω*_31_, *S*_0_(*ω* − *ω*_21_) and *S*_0_(*ω* − *ω*_31_), in Fig. 2(a) and (d) are measured in the absence of the control pulse, and the line shape is thus Lorentzian at each transition. The red dashed lines show the simulated absorption spectra without modulation, which agree well with the measurement. These spectra are employed to extract the prefactors (*d*_1*k*_*ρ*_1*k*_(0)), which are subsequently used to fit the time-delay-dependent absorption spectra via Eq. 1 and, thereby, extract the control parameters in Eq. 2. *ρ*_1*k*_(0) are the density-matrix elements generated after the interaction with the weak probe pulse. In the simulation, we do not use the theoretical values of *d*_1*k*_ explicitly, as *d*_1*k*_*ρ*_1*k*_(0) can be directly extracted from the spectra in the absence of a control pulse [*d*_12_*ρ*_12_(0)/(*d*_13_*ρ*_13_(0)) = 1.147]. The exact absolute number of the dipole moments is not important, since the spectra are presented in arbitrary units and normalized to the same common factor. By eliminating the broadening due to the control pulse, the decay rates are set to be Γ_2_ = Γ_3_ = 1/6 ps^−1^ to compensate the Doppler broadening and collision-induced broadening.

When the control pulse interacts with the excited quantum system, the dipole responses will be modified in the time domain both in amplitude and phase. The time-dependent interaction of the three-level system with intense short pulses induces Rabi oscillations in the populations of the three states. The excited states 5p ^2^P_1/2_ and 5p ^2^P_3/2_ can also be further coupled to higher excited levels or to the continuum via strong-field ionization by the control pulse. This results in a transformation of the state of the three-level system from an effective initial state (preceding the interaction with the intense control pulse) into an effective final state (following this interaction), which will then evolve freely as a result of the atomic-structure Hamiltonian^33^. The effective final state can be related to the effective initial state via the transformation modeled by the DCM. The 8 parameters of the two time-delay dependent functions *A*_*k*_(*τ*) are used to quantify this transformation from the transient-absorption spectrum. Figure 2(b) and (e) show the time-delay-dependent absorption spectra with a control-pulse intensity of *I*_co_ = 2.13 × 10^10^ W/cm^2^. The absorption line shapes at both transitions show a periodic dependence upon the time delay *τ*, where the period is given by the beating frequency *f*_b_ = 2*π*/*ω*_32_ = 140.43 fs. The two absorption lines *S*(*ω*_21_, *τ*) and *S*(*ω*_31_, *τ*), centered at the transition energies *ω*_21_ and *ω*_31_, oscillate in time delay *τ* with frequency of *f*_b_. Similar oscillations were also detected when the control pulse precedes the probe pulse^24^,^34^. The fast oscillations in frequency of the experimental results [see, e.g., Fig. 2(a) and (d)] are caused by artefacts in the electronic readout of the spectrometer, as we checked by comparing spectrum of the laser pulse without interaction.

The inter-state processes are responsible for temporal oscillations corresponding to the energy difference of the two excited states, as opposed to the self-processes for which they are absent. By fitting the DCM to the experimental data, the modulation parameters are extracted and estimated to be 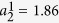, 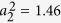, 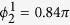, 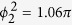, 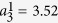, 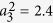, 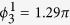, 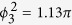. The amplitude parameters 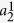 and 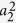 can quantify the weights of the self and inter-state contributions to the dynamics of the 5p ^2^P_1/2_ state due to the control pulse interacting with the multi-level system, while 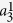 and 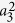 quantify contributions to the dynamics of 5p ^2^P_3/2_ state. With these parameters, the calculated transient-absorption spectra *S*(*ω* − *ω*_21_, *τ*) and *S*(*ω* − *ω*_31_, *τ*) are presented in Fig. 2(c) and (f), respectively. The decay rates of both excited states are set to be Γ_2_ = 1/3.5 ps^−1^ and Γ_3_ = 1/2.2 ps^−1^ in order to approximate the experimental line widths, much larger than the spontaneous decay rate. Here and in the following, the scaling of these amplitudes and of the simulated absorption spectra is by arbitrary units.

**Figure 3:**
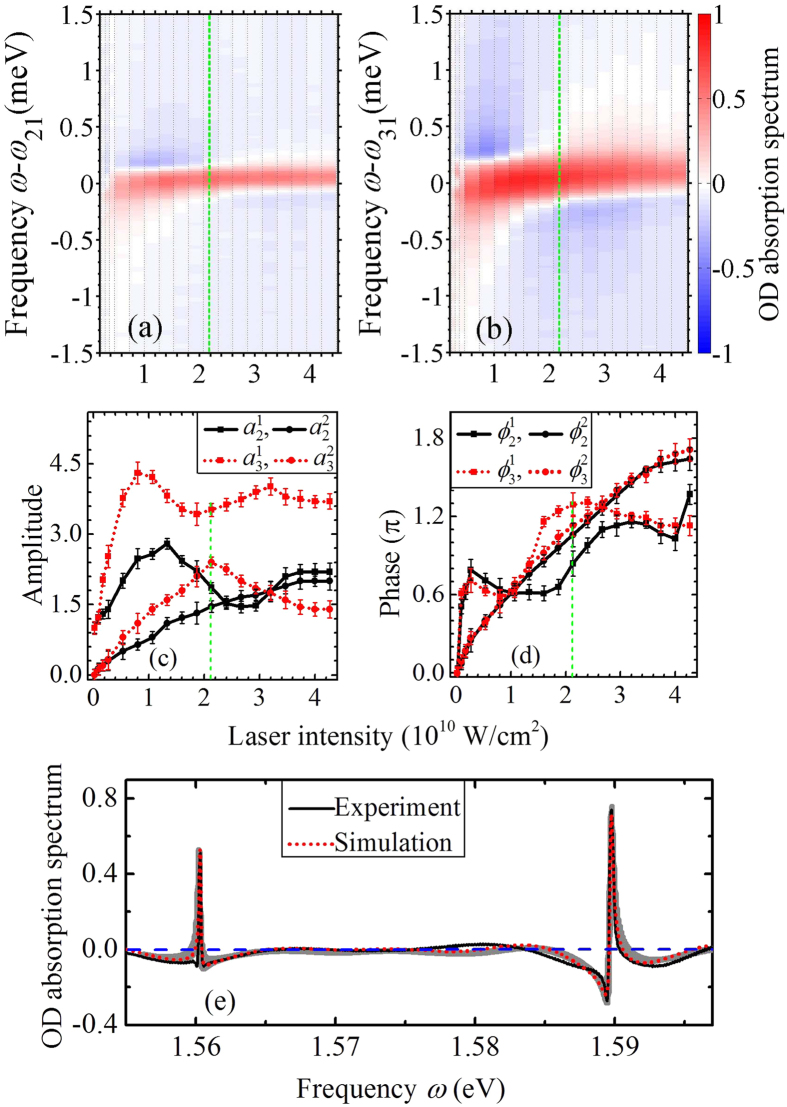
Experimental absorption spectra *S*(*ω* − *ω*_21_) and *S*(*ω* − *ω*_31_) at frequencies (**a**) *ω*_21_ = 1.56 eV and (**b**) *ω*_31_ = 1.59 eV of a probe pulse of intensity *I*_pr_ = 3.0 × 10^8^ W/cm^2^, transmitted through an ensemble of Rb atoms interacting with a fixed-delay (440 fs) control pulse of varying intensity. Results lined out by the vertical dashed lines in (**a**) and (**b**) correspond to the experimental absorption spectrum that shown in (**e**). Panels (c) and (d) show the amplitude and phase parameters extracted from the time-delay-dependent absorption spectra of varying control-pulse intensities, in which the vertical dashed lines show these parameters that used to generate the simulated result in (**e**). Results in (**e**) show the experimental and DCM-fitted absorption spectra with a fixed control-pulse intensity of *I*_co_ = 2.13 × 10^10^ W/cm^2^, and the horizontal dashed line indicates the zero.

To study strong-field effects, in the following we focus on the dependence of the amplitude and phase modulation of the spectra on the control-pulse intensity. Figure 3(a) and (b) display the measured absorption spectra around both transition energies *ω*_21_ and *ω*_31_, respectively, at a fixed probe-control delay of 440 fs for varying control-pulse intensities. The maximum intensity that can be applied is limited by the control pulse filamentation in the front window of the Rb gas cell. The line widths are observed to vary as a function of intensity and are hence taken into account by free fit parameters. The influence of the control pulse on the quantum system can be quantified by applying the DCM, and the intensity-dependent modulation parameters 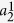, 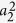, 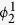, 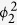 for the state 5p ^2^P_1/2_ and 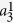, 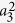, 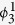, 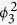 for the state 5p ^2^P_3/2_ are extracted and shown as functions of the control-pulse intensity in Fig. 3(c) and (d). Results in Fig. 3(e) display the measured and simulated absorption lines. The gray region behind the simulation and experimental curves shows the possible range of the absorption line implied by the error bars of these 8 parameters, from which one can find the results obtained by the DCM agree well with the experimental result. For the lowest control-pulse intensities considered, two Lorentzian absorption lines centered on the transition energies are obtained. The amplitude parameters for the self contribution 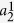 and 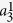 are 1.0, while the parameters for the inter-state contribution 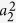 and 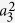 are 0. With the increase in the control-pulse intensity *I*_co_, a competition is displayed between self (amplitude parameters 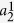 and 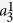, for the state 5p ^2^P_1/2_ and 5p ^2^P_3/2_, respectively) and inter-state processes (amplitude parameters 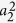 and 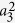, for the state 5p ^2^P_1/2_ and 5p ^2^P_3/2_, respectively) as a function of the control-pulse intensity. The different resonant coupling of the two excited states to the ground state or other surrounding states results in the evolution of these amplitude parameters. Phase parameters for varying control-pulse intensities are exhibited in Fig. 3(d). With the lowest control pulse, the phases 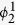 and 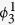 are 0*π*, indicating that weak pulses do not affect the system. The phase parameters 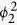 and 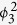 due to the inter-state process increase with the control pulse intensity *I*_co_.

The experimentally reconstructed DCM parameters in Fig. 3(c) and (d) allow one to quantify how the state of the system is transformed by a strong control pulse, and in particular how the two coherences mutually affect each other. Although the DCM does not provide access to the time evolution of the coherences, it enables one to access how the control pulse transforms the system, in amplitude and phase, from an initial state into an effectively final state, respectively before and after interaction with an intense pulse. The behavior displayed by the amplitudes at low control-pulse intensity clearly shows that single-photon self-processes are dominant, with the two coherences only marginally influencing each other. The appearance of intensity-dependent Rabi oscillations of populations manifests itself in two ways. On the one hand, we notice a decrease in both 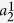 and 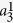, i.e., a decrease in the final coherences after the interaction with the control pulse. This could be related to the fact that the intense control pulse, as a result of Rabi oscillations, has moved populations from the excited states back to the ground state, reducing the associated resulting coherences *ρ*_1*k*_. On the other hand, we notice an increase in the DCM parameters of inter-state multiphoton processes, with the initial population in one excited state increasingly influencing the final population in the other excited state. With transient-absorption spectroscopy the information on this intensity-dependent transformation can be extracted in amplitude and phase.

## Conclusions

In conclusion, we have used a V-type three-level system, which was first excited by a probe pulse and subsequently modified by a control pulse, to study the amplitude and phase modification of a coherently excited quantum state, imprinted as the change of the absorption spectral line shapes of the probe pulse. The DCM is used to interpret these absorption spectral line changes, based on eight modulation parameters extracted from the time-delay-dependent absorption spectra. We demonstrate that the DCM parameters can be used to model the transformation of a multi-level system in the presence of an intense control pulse. The amplitude parameters 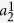 and 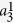 characterize the self contributions of the control pulse to the dipole responses, while 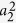 and 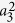 represent the inter-state contributions. The dependence of the transient-absorption spectra upon the control-pulse intensity indicates that the quantum dynamics can be manipulated by strong-field effects by tuning this intensity, taking advantage of the different coupling of the two excited states to the ground state and other surrounding levels. The intensity-dependent amplitude and phase modulation allows us to quantify the quantum dynamics of an excited atomic system, which can also be further transferred to more complex systems such as molecules and nanoparticles.

## Methods

A commercial Ti:Sapphire chirped-pulse amplification laser system operating at 1 kHz is employed, providing laser pulses with pulse energy of 0.8 mJ, pulse duration of 30 fs and center wavelength of 800 nm, which is transform-limited. As shown in Fig. 1(b), the laser beam is split by a spatial mask with two irises into two beams, which are used as probe and control beams, respectively. A delay line is included into the control beam path, allowing one to scan the probe–control pulse time delays. The probe and control pulses are focused by a concave mirror and the sample is set in front of the focus, with beam sizes of 1.3 mm and 1.0 mm for the control and probe beams in the interaction region, respectively. After passing through the sample, the probe beam is picked up by an identical concave mirror and coupled into a fiber-pigtailed spectrometer (McPherson 2061). A CCD camera monitor allows one to check the temporal and spatial overlap of the pulses at the focus during the measurement, as it provides a real-time image at the focus. The control-pulse intensity can be tuned by using a continuously variable neutral density filter in the control beam path right after the mask. A rubidium gas cell (Thorlabs) with a length of 20 mm is used as sample, heated to 150 ± 0.2 °C by a homemade heating and control system. The Rb atomic density was estimated to be 1.9 × 10^14^ cm^−3^ at this temperature^35^.

## Additional Information

**How to cite this article:** Liu, Z. *et al*. Observation and quantification of the quantum dynamics of a strong-field excited multi-level system. *Sci. Rep.* **7**, 39993; doi: 10.1038/srep39993 (2017).

**Publisher's note:** Springer Nature remains neutral with regard to jurisdictional claims in published maps and institutional affiliations.
